# The sociometry and sociogenesis of reproduction in the Florida harvester ant, Pogonomyrmex badius

**DOI:** 10.1673/2006_06_32.1

**Published:** 2006-10-23

**Authors:** C. R. Smith, W. R. Tschinkel

**Affiliations:** 1 Dept. of Biological Science, Florida State University, Tallahassee, FL 32306-4370; 2 Current Address: Program in Ecology and Evolutionary Biology, Dept. of Animal Biology, University of Illinois, 515 Morrill Hall, 505 S. Goodwin Ave., Urbana, IL 61801

**Keywords:** Pogonomyrmex, reproduction, growth, fitness, allocation, allometry

## Abstract

The colony is the functional unit of natural selection for most social insects including the Florida harvester ant, Pogonomyrmex badius. To address reproduction in the species variables were evaluated relevant to the colony-level (sociometry), and social growth (sociogenesis). Colonies become reproductively mature when the worker population reaches ~700 individuals. The production of males and gynes (reproductive females) occurs only in spring, and is highly synchronized from its onset, which in turn allows synchronization of mating in early summer. Worker production follows sexual production, and continues until colonies go dormant in winter. Once mature, colony investment into reproduction is a constant proportion of colony size (isometric), regardless of the sex ratio produced. As individual male body size increases, they become leaner, whereas the amount of fat stored by gynes is highly variable. Larger colonies produce larger males, but gyne size is a constant across the range of colony sizes. As colony size increases, investment into males increases faster than investment into gynes. Therefore, there is a trend of increasing male bias in the sex ratio with increasing colony size (although a single outlier complicates this conclusion). We interpret our results in the light of sexual and natural selection as documented in related species. We also report the first documentation of male production by workers in the genus Pogonomyrmex.

## Introduction

In eusocial organisms, selection acts on the collective unit (colony), rather than individuals (Bourke and Franks 1995; Korb and Heinze 2004). Ultimately, natural selection favors strategies that maximize the number of new colonies successfully established. The manner in which new colonies are founded and grow differ tremendously in the social insects, and ants run the gamut from independent (a solitary foundress) to completely dependent (i.e., budding) colony founding (Holldobler and Wilson 1990).

Harvester ants of the genus Pogonomyrmex have a well-described mating system that is analogous to broadcast spawning. Colonies send out males and gynes (reproductive females) in highly synchronized nuptial flights (Strandtman 1942; Michener 1948, 1960; Van Pelt 1953; Nagel and Rettenmeyer 1973; Clark and Hainline 1975; Holldobler 1976; Davidson 1982; Mintzer 1982; Rust 1988; Harmon 1993; Cole and Wiernasz 1997). The sexual individuals, once released from the parent colony, increase their chance of successful fertilization by aggregating at leks (see Holldobler 1976). Once mated, the queen founds a new colony (different species may found colonies solo or cooperatively: Johnson 2004). Parent colonies can better their odds of producing a successful founding queen or the male(s) that inseminated her using a variety of strategies, at a variety of levels. For example, by modifying the size or fat reserves of individual males and gynes, a parent colony can produce higher quality reproductives that are more likely to succeed at colony founding (examples from Pogonomyrmex occidentalis: Abell et al. 1999; Wiernasz et al. 2001;Wiernasz and Cole 2003). At the colony level they can alter the sex ratio and total numbers of individuals produced (see review by Mehdiabadi et al. 2003), as well as changing reproductive timing, and the duration of reproduction (i.e., the number of mating flights in which they participate). If colonies are able to reproduce in multiple years, they may be able to augment lifetime reproductive output by increasing the size of their worker population because larger colonies have higher reproductive potential in Pogonomyrmex (Mackay 1981; Cole and Wiernasz 2000; Tschinkel 1993). Therefore, to obtain maximal fitness, colonies must balance investment into growth and reproduction, and then further modify their investment in reproduction in a way that maximizes their genes in the next generation (Stearns 1992; Bourke and Franks 1995).

In this paper the Florida harvester ant (Pogonomyrmex badius), was used to explore the ontogeny (sociogenesis) of reproduction at the colony level, and the strategies that may maximize fitness across colony sizes. Our knowledge of this species’ seasonal variation and sociogenesis (Tschinkel 1998, 1999a,Tschinkel b) allow us to investigate in detail how selection has shaped colony investment in growth and reproduction.

The genus, Pogonomyrmex, is well studied and ecologically important (Taber 1998; Johnson 2001), but colonies are often inaccessible because they typically nest in rocky soils, limiting our knowledge of the colony-level processes underlying ecological and evolutionary questions. Because P. badius lives in deep sandy soils, we are able to excavate whole colonies to examine how they invest in both reproduction and growth.

This paper addresses the following questions about allocation to reproduction: 1) what is the timing of growth and reproduction during the annual and lifetime cycle? 2) what are the patterns of resource investment in reproductive individuals, at both the individual and colony-level? and 3) how does colony ontogeny affect sexual allocation?

### Site description, colony excavation, and measurements

All colonies were collected in the Apalachicola National Forest, about 15 km southwest of Tallahassee, Florida. Nineteen colonies were excavated in an area that was clear-cut in 1999 and planted with longleaf pine, few of which were taller than 1 m at the time of this study. Ground cover vegetation was shrubby and herbaceous, predominantly shiny blueberry (Vaccinium myrsinites). Due to the clear-cut and the lack of P. badius in the surrounding forest, it is likely that none of the study colonies was greater than 4 years old.

Mature, reproductive, colonies were selected by mound size, a good proxy for colony size (Smith 2004). Reproductive colonies were of two experimental treatment groups, with their charcoal covering removed or removed and replaced (n = 9 in each group). The experiment was designed to test whether fitness was affected by charcoal on top of the mound (large nests can have 1/3kg of charcoal, ~300,000 pieces). Harvester ants are notorious for collecting objects, including charcoal, which are deposited on top of their nest Smith and Tschinkel 2005). This experiment addressed the adaptive potential (affect on reproductive fitness) of objects on top of the nest. Because no effects of charcoal were detected (Smith 2004), data were pooled across the treatments for the analysis presented here (17 of 19 excavated colonies produced sexual offspring).

Colonies were excavated with a shovel as in Tschinkel (1998), where an initial hole (pit) was dug adjacent to the colony, and chambers were exposed sequentially from the top down, working from the pit. For completeness, soil was excavated either 25cm deeper than ants were found or to the water table.

Ants were brought back to the lab, frozen, sorted and counted. All larvae were thought to be workers, based on their size when excavated. In their final instars sexual larvae are much larger than those of workers. After counting them, ants were dried at 60°C in an oven for at least 48 hrs, and weighed to the nearest milligram. A sample of 10 males and 10 gynes were individually weighed on an electronic microbalance (to the nearest mg for gynes, and tenth of a mg for males), and their head widths estimated by measuring the inter-ocular distance. Fat was extracted using ethyl ether as the solvent in a Soxelet extractor for at least 24 hours. The energetic content of individuals was calculated based on the conversion values of Peakin (1972) for fat and lean mass (fat = 39.33 J/mg; lean = 18.87 J/mg).

### Reproductive schedule

The relationship between the collection date of a colony and the proportion of pupae and callows in the nest was examined to evaluate reproductive synchrony across colonies. If this relationship is linear then colonies are synchronized in their production schedules, all having started producing at about the same time. To estimate the time at which colonies began production the pupation rate was extrapolated to 100% pupae, and egg, larval and pupal times estimated from the literature. This information is not known for P. badius, so data was used from other species (Porter 1988). To verify the estimate of reproductive onset three colonies were excavated, noting the presence or absence of brood in each. The first was on 11-March, 2004 (C7), the second on 25-March (C25), and the third on 15-April (C56). Both March colonies were returned to the field after assessment, while C56 was retained as a lab colony because the queen was accidentally decapitated during excavation. This colony was used to assess the possibility of worker reproduction in the absence of their queen. Workers were divided into 2 sub-colonies of approximately equal size and casually observed for 3 months.

### Individual-level size and allometries

To assess how total mass and fat content changed relative to individual size (approximated by head width) individuals were pooled from all colonies.

To confirm the assumption that dry weight is a good approximation of the energetic cost of manufacturing (excluding maintenance cost – respiration – of individuals) dry weight and energetic content of both workers and reproductives was regressed. Boomsma (1989; Boomsma et al. 1995) demonstrated that using manufacturing cost overestimates the energetic cost of females in species with a sexual size dimorphism (due to respiration costs), and proposed correcting mature adult dry weights using a power conversion of 0.7. Comparisons of estimates of sex ratio (expressed as the proportion female) were made using the manufacturing cost and the transformed adult dry weight estimate (x^0.7^) using a t-test.

Whether gyne fat content was affected by the date of collection (since adult gynes accumulate large fat reserves prior to flights) was examined by regressing dark (mature) gyne proportion fat against the collection date. A positive slope would indicate a sampling bias.

### Colony-level allometries

Colony size, the number of mature workers in the nest, was used as the independent variable for all allometric analyses. The dependent variables are estimates of sexual production, represented by their weight or the number of individuals. Weights are sensitive to when colonies, especially those sampled in spring, are excavated because they have differing proportions of ants in each developmental state, and adults weigh more than pupae. Therefore, the end-weight of workers and sexuals was estimated to account for sampling effects. Numerical sexual production was the sum of sexual pupae and adults of both sexes, and by weight was the number of males multiplied by the average male weight from that colony, plus the number of gynes multiplied by the average gyne weight across colonies (some colonies did not have mature gynes).

### Statistical methods and calculations

Variables were transformed to satisfy assumptions of statistical tests, using a log_10_ transformation on most variables, but arcsine transformation on proportions. Allometries were done using Type-I regression because it is a standard method employed for such analyses, though we recognize that Type-II regression is the more appropriate statistical tool. Isometry (slope different than 1) was tested using a t-test. Individual allometries used data pooled from all colonies, whereas colony allometries used the average or total per colony. All allometries of gynes are of dark individuals (presumed mature). All analyses were done in Statistica 6.0 (Statsoft 2003).

## Results

Seventeen of the 19 colonies reproduced during this study. All reproductive colonies had greater than 700 workers. A queen was collected in 18/19 colonies, producing a high level of confidence that excavations were complete. The Appendix contains a simplified version of the entire dataset.

### Production schedule

**Figure 1:**
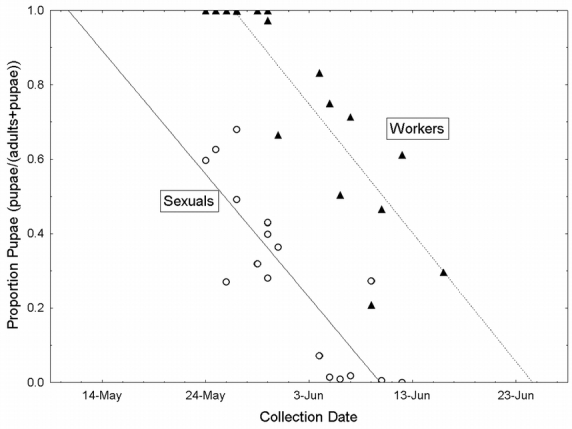
The pupation rate of workers and sexuals, represented as the proportion pupae (of pupae and adults) over the collection dates of colonies. Open circles and the solid line represent reproductives, while closed triangles and the dashed line are workers.

Colonies were highly synchronized in productive scheduling. The proportion of both reproductive and worker pupae decreased linearly with date, such that date explained 87% and 71% of variance, respectively. Both sexual and worker adults emerged from pupae at a constant rate of about 3.6% per day (slopes were 0.37 and 0.35 respectively, both slopes were different than 0, F_1,15_ = 98.7 and 36.7, in both cases p < 0.0001). Furthermore, slopes of worker and reproductive pupation rates did not differ from each other (t_0.05[32]_ = −0.29, p > 0.5). Reproductives, however, began emerging 14 days before workers (Fig. 1). Therefore, sexual production was a discrete pulse, with worker production following.

**Figure 2:**
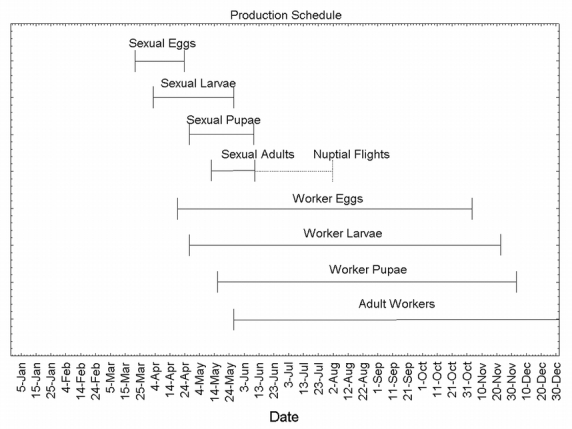
The production schedule of P. badius. Horizontal bars represent time when that developmental stage is predicted to be in the colony. The dashed line represents the approximate time span of nuptial flights.

To make an estimate of when each phase of production began, data were used that had been published on other ant species. This was only a qualitative estimate. Data on workers of 7 ant species reviewed by Porter (1988) showed an average developmental time of 38 days (from egg to adult), at an average temperature of 27°C. A 38-day development time was assumed for P. badius workers, and 50 days for reproductives. Furthermore, the proportion of time spent in each developmental state was assumed to follow that of fire ants, or 1:2.4:1.3, egg to larva to pupa, independent of temperature (Porter, 1988). These estimates yield the following breakdown of time in each developmental state (egg-larva-pupa): reproductives, 11-25-14 days, and workers, 8-19-11. In this forecast the effect of temperature on development was ignored due to the lack of information regarding the relevant temperatures experienced by the developing brood inside the nest. Figure 2 is the projected developmental schedule for all production in a P. badius colony. These calculations indicate that P. badius starts producing sexual eggs at the beginning of April and worker eggs around mid-April. Tschinkel (1999a) found that worker production continues through October, but no immature stages were found in colonies during January.

No brood was found in the colonies excavated in March, despite these colonies being of mature size (C7 = 7524 and C56 = 6815 workers). Colony C70, excavated on 15-April had 7853 workers and 2 larvae (eggs were not systematically collected, and hence not counted). This supports the accuracy of the estimates of production, as larvae were forecast to be present on 3-April in a reproductively mature colony. The recovered larvae from C70 matured in the lab and were identified as gynes at the pupal stage. Interestingly, workers killed the gynes before they became adults. Workers from both halves of C56, the queen-less colony, produced males. Male production was obviously by workers because it continued over 3 months after the colony was accidentally de-queened.

The development schedule also supports the assumption that the larvae found in the 19 colonies were not sexual. The forecast (Fig. 2) suggests that sexual larvae were no longer present by 25-May, the day after the main bout of excavations started.

### Individual-level size and allometries

Gynes were 1.5 times as large as males (by head width), 3.6 times as heavy (by dry weight), 3.4 times as fat (proportion fat), and 4.6 times more energetically expensive (Table 1). Male weight increased with size, but the proportion of fat decreased (Table 2). For every 10-fold increase in size (head width) there was a 14-fold increase in weight, but only a 3-fold increase in fat. Gyne allometries were less clear. Gyne weight was isometric with size, but size only explained 9% of the variance in weight. No relationship was found between size and fat reserves in gynes. Collection date was not a significant predictor of gyne fat reserves (as a proportion of dry weight; F_1,13_ = 0.06, n.s.), indicating no sampling bias for gynes. Furthermore, the fat content calculated for gynes (37%) was similar to calculations on congeners with similar founding strategies (~40% fat by weight; Hahn et al. 2004).

**Table 1:**
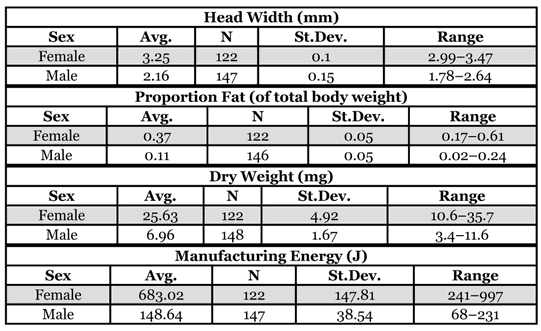
Breakdown of male and gyne size, weight, fat content, and manufacturing cost. Data are pooled from all colonies.

**Table 2:**
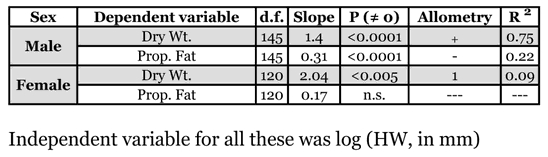
Male and gyne body allometries. The independent variable, body size, is approximated by head width (in mm). Data are pooled from all colonies. All variables were log_10_-transformed.

On an individual basis, gynes cost colonies over four times as much as males. Adjusting the manufacturing costs (biomass) of reproductives using the correction suggested by Boomsma (1989), which was expressed as biomass^0.7^, energetic sex ratios (proportion female) were 0.54 +/− 0.28 (unadjusted) and 0.46 +/− 0.26 (adjusted using the Boomsma correction) (t_0.05[29]_ = −0.79, n.s.). The power adjustment of biomass suggested by Boomsma helps account for size-specific metabolic costs, where larger individuals expend less energy due to metabolism, per their size, compared to smaller individuals.

The relationship between dry weight and energetic content, across all individuals (minor and major workers, males and gynes), fit the data very well (r^2^ = 0.99; F_1,864_ = 69,874, p << 0.001) with a slope of 1.09. This indicates that the use of dry weight to approximate energetic cost was valid.

### Colony-level allometries

**Figure 3:**
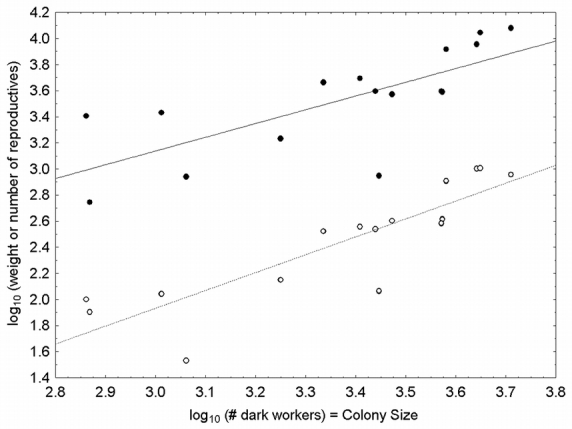
Weight and numeric allometries of reproductive production. Filled symbols (with solid line) are weights and open symbols (with dashed line) numbers. The independent axis is the number of mature workers in a colony (colony size). The slope (scaling exponent) of the weight-colony size relationship is 1.06 and for numeric data is 1.37. Neither slope is statistically different than 1.

Investment into sexuals, by both weight and number, was isometric with colony size (Fig. 3). Colony investment into individual male qualities (head width, dry weight, and fat content) increased more slowly than colony size (Table 3). Gyne qualities, however, were not associated with colony size at all. Total production of males, the number produced and their summed weight, increased faster than colony size. Total gyne production, numerically, increased at the same rate as colony size, but their summed weight was not associated with changes in colony size (Table 4). Therefore, colonies invested proportionally more in total male production by increasing both the total number produced and the quality of individuals. On the other hand, investment into gyne quality was constant, regardless of colony size, but the total number produced increased proportionately to colony size.

**Table 3:**
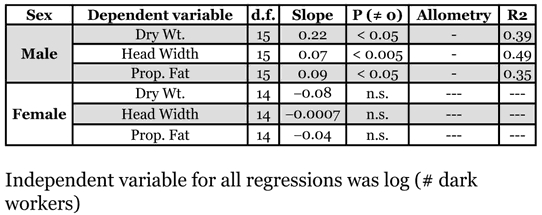
Individual male and gyne colony-level allometries. The independent variable is the number of mature workers in a colony (colony size). All dependent variables are derived from colony averages. All variables were log_10_-transformed.

**Table 4:**
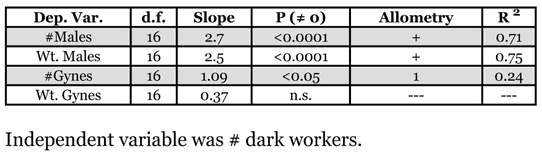
Energetic and numeric allometries of male and gyne production. The independent variable is the number of mature workers in a colony (colony size). All variables were log_10_-transformed.

**Figure 4:**
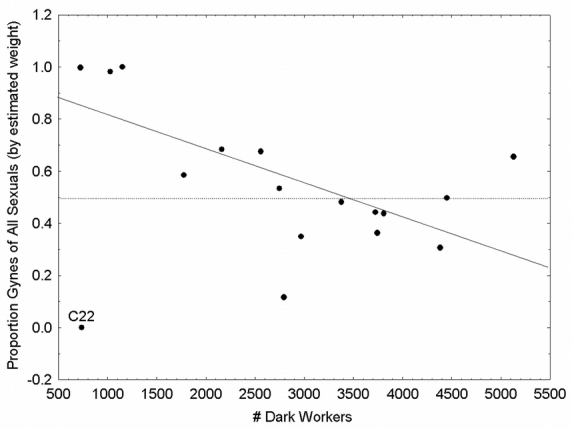
Sexual allocation, expressed as the proportion invested into gynes (by estimated dry weight) against colony size (the number of mature workers). The dashed line represents equal investment in both sexes while the solid line is the fit from a linear regression excluding C22, statistically an outlier.

The interpretation of the sex ratio in these colonies is dependent on the inclusion or exclusion of a single colony, C22, which was statistically an outlier. Both interpretations are presented because it was uncertain whether this colony was biologically anomalous. C22 was one of the smallest colonies (738 mature workers), and produced an all male reproductive brood. It also produced workers, indicating the queen was inseminated. If C22 is included, colony size and sex ratio are unrelated (Fig. 4). The only detectable pattern was high variability in small colonies, with larger colonies stabilizing at 53% female production by weight and 33% numerically. Smaller colonies tended to produce heavily sex-biased broods. Colonies under 1500 workers (n = 4) all had a bias of 90% or more, 3 female biased and 1 male biased. Colonies with over 1500 workers average 47% +/− 0.16 gynes by weight, 22%+/− 0.10 numerically. If C22 is excluded, there is a significant decrease in gyne investment by weight as colonies increased in size (F_1,14_ = 11.0, p < 0.05)(Fig. 4).

## Discussion

### Production schedule

Production in this species is highly synchronized among colonies, as would be expected, since nuptial flights (i.e., mating) are somewhat unpredictable events, usually following the first heavy summer rain. Furthermore, successful flights require the participation of multiple colonies, or colonies risk sib-mating (which occurs at high levels in P. occidentalis, Cole and Wiernasz 1997). Therefore, all colonies have some of both sexes ready to fly when the first rain comes, but continue to rear more for subsequent flights. This reproductive synchrony is likely achieved through a cue that initiates production at the beginning of spring. Knowing the production schedule of this species facilitates experimental work by providing an estimate of what is in the colony at any given time, and when colonies switch from their annual reproductive- to worker-producing phase.

Observations made on a single queenless colony suggest that multiple workers within a nest will become reproductive when the queen is removed. Although uninseminated, the workers can still produce males and potentially gain in fitness. This is relevant to sexual allocation because it cannot be assumed that all male production is from the queen. When a queen is removed from a nest the ovaries of workers become more active (have more developed oocytes - Smith, unpublished data), making it likely that the queen inhibits male production by workers. To our knowledge, this is the first report of male production by workers in the genus Pogonomyrmex.

### Individual-level size and allometries

There was a pronounced sexual dimorphism in this species, with females much larger and more expensive than males. The energetic cost of manufacturing individuals (excluding respiration costs) is well predicted by dry weight, justifying the use of dry weight as a proxy for energetic investment (colony-level allometries). Furthermore, accounting for respiration costs as suggested by Boomsma (1989) did not significantly change the ratio of investment between the sexes, and both are not different than 1:1. Therefore, results were interpreted using only dry weights.

There are pronounced allometric relationships of male characters. As males increase in size they increase proportionately in weight, but become leaner. P. badius queens are known to mate with many males (Rheindt et al., 2004) in the mating swarms (leks), so that male size may be very beneficial in obtaining a mate (Davidson 1982; Abell et al. 1999; Wiernasz et al. 2001). As males increase in size they are not putting on extra fat reserves, but simply increasing in lean mass. Increased lean mass may be advantageous in male-male competition, or perhaps dispersal. Male size is often correlated with increased sperm transfer (e.g.,Wiernasz et al. 2001), which is in turn the best paternity estimate in the honeybee, another highly polyandrous hymenopteran (Schlüns et al. 2004). Therefore, it would seem likely that larger males are able to obtain more fitness for the colony. Gynes do not increase in weight or fat content as a function of size, and there is less variation in gyne size. Natural selection may work to decrease variance in gyne size by 1) putting a minimum on size and fat stores for successful colony founding (e.g., Wiernasz and Cole 2003), and 2) putting a maximum on size due to their high manufacturing cost, possible diminishing returns of fitness, or a physiological size limit.

### Colony-level allometries

Interpretations at the colony-level demonstrate that patterns of investment into the sexes change as a function of colony growth. Individual male size and fat content increased more slowly than colony size, but larger colonies did produce larger males (Table 3). Furthermore, as colonies grew they invested disproportionately more into males, increasing male production 20-fold for every 10-fold increase in colony size (Table 4,Figure 4). On the other hand, gyne size and fat content were independent of colony size. And though the number of gynes produced by colonies was a constant function, total investment into gynes (by weight) was independent of colony size.

This pattern suggests that there is a fitness benefit to producing more and larger males. Sexual selection on male body size (Abell et al. 1999), as well as assortative mating (Davidson 1982), are known from other Pogonomyrmex species. This result is not too surprising given the lek mating system of most species (reviewed in Holldobler 1976), and although the mating biology of P. badius is not well understood, mating aggregations at least occur at nests (Harmon 1993; and personal observations).

The interpretation of the sex ratio changes with the inclusion of a single colony, C22. If C22 is included, the average sex ratio (proportion female, dry weight) is approximately 1:1, as expected from the genetic relatedness hypothesis (Trivers and Hare 1976; Nonacs 1986). According to this hypothesis, as the queen mates with more and more males, the sex ratio converges on a 1:1 sex investment. As colony size increased, the variance in the sex ratio decreased, until converging at ~50% female. If C22 is excluded, there was a significant negative slope to the relationship. If this result is not an anomaly, two hypotheses are suggested that could explain this result: 1) given the genetic relatedness hypothesis, worker relatedness within the colony may decrease with colony growth due to an increase in the number of patrilines among the workers, resulting in decreased female investment, or 2) the fitness gains of males and females are different, with male reproductive value increasing faster than that of females, in both quality and quantity (Frank 1987). Pogonomyrmex badius are only known to found colonies haplometrotically (by a solitary foundress), and mature colonies always have a single queen (personal observations of both authors). Therefore, if hypothesis 1 were true, sperm precedence would be the likely mechanism for changes in relatedness through time, and not multiple queens. Queens of P. badius mate with an average of 9–10 males, and up to a maximum of 29 (Rheindt et al. 2004), but no information is available regarding sperm precedence in harvester ants. Little information exists on sperm precedence in most social Hymenoptera, and has been best studied in honeybees, Apis mellifera. Schluns et al. (2004) report a marginal effect (P = 0.08) of insemination sequence in predicting patrilines of honeybee workers born from artificially inseminated queens. In the same study, most variation in patriline representation among workers was explained by the volume of inseminate, not order of insemination. On the other hand, various theories make predictions about differential fitness returns between and among the sexes, which would support hypothesis 2.

Frank (1987) reviews several theories to predict non-linear fitness returns within a sex. Local resource enhancement predicts a synergism between related individuals of the same sex, as in cooperatively founding queens, which is not relevant in P. badius. Local resource competition and local mate competition both predict a diminishing return in colony fitness due to antagonisms between related individuals of the same sex, due either to resource (e.g., access to nest sites or food for gynes) or mate competition, which is more likely in males. The results of this study, where male investment increases much more rapidly than gyne investment, are not consistent with any of these scenarios unless related males act synergistically (i.e., local resource enhancement, where resources are presumably mates), which would seem unlikely. Multifaceted parental investment (MFPI)(Rosenheim et al. 1996) makes predictions for the variation of investment within sexes, where variation in individual size is due to a trade-off between resource limitations (e.g., brood/egg production or energetic limitation) and optimal offspring size. In the context of MFPI, male fitness returns are highly variable and vary with male size, where larger males presumably yield greater fitness for the colony, but gyne fitness is constant and likely constrained (see above discussion of individual gyne size). If male fitness returns are highly variable, even when producing large males, colonies with few resources may be expected to invest proportionally more resources in gynes, such that they minimize the variance in expected fitness returns.

Sex ratios are highly variable both spatially, within and between sites, and temporally, between seasons, (Elmes and Wardlaw 1982; Elmes 1987; Herbers and Banschbach 1998). Furthermore, hypotheses that attempt to explain sexual allocation in the hymenoptera are numerous, and may or may not differ regarding their predictions (see review by Mehdiabadi et al. 2003). The results and theoretical considerations of others suggest that it is best to remain skeptical about the robustness of this pattern, and collect more data in order to test particular hypotheses about sexual allocation in this species. This same skepticism applies to our entire analysis of reproduction because it draws on information from only a single year and site. Our argument for the validity and generality of the information presented is simple: the robustness of most patterns we present at least indicates our ability to understand production at the sites where it is described, and if we assume that patterns of colony development are heritable, our analysis is not likely to stray far from the general trends about this species and even genus.

In summary, colonies begin their reproductive phase of life when they reach a mature worker population of ~700. In early spring (the end of March in this population), the colony begins production of males and gynes, and switches to worker production approximately a month later. Worker production continues until the beginning of winter (December in this population) when the colony becomes reproductively inactive (December to March). As colonies increase in size they are capable of producing more reproductives, and workers. Although the production of both males and gynes increase with colony size, male production increases twice as fast. This asymmetry yields a changing sex ratio as colonies grow, whether variance decreases or the slope is negative. Bigger colonies also produce bigger males. This likely increases the colony’s probability of fitness by gaining a numerical advantage over neighboring colonies, and also by producing better males (if size does indeed matter). This is not, however, the case with gyne production. All colonies produced approximately the same size of gyne, and this size is possibly the result of selective pressures to both decrease maximum size and maintain a minimum size. The fitness of a colony is the number of new colonies in the next generation, summed over all of the breeding seasons in which the colony participated. Selection favors colony growth because growth creates the largest increase of reproductive output. Colony fitness returns are possibly non-linear with size due to larger colonies’ ability to produce not only more males and females, but also larger males. Alternately, it is possible that fitness returns are constant, but the optimal investment strategy differs as a function of the amount of energy a colony has available to invest. Differing investment strategies may be represented by the sizes of males and gynes, and the total number produced of each.

## Figures and Tables

**Appendix A:**
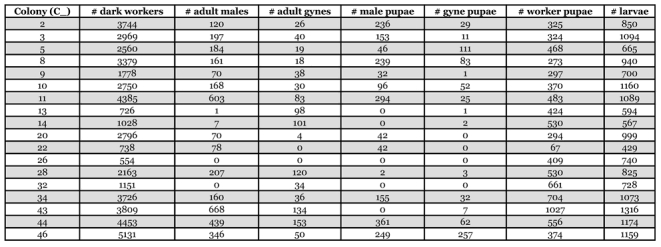
A simplified version of our dataset
